# Six year natural progression of a clinically monitored retroperitoneal schwannoma: A case report

**DOI:** 10.1016/j.ijscr.2024.110622

**Published:** 2024-11-16

**Authors:** Elizabeth Kruse, Allye Gardner, Eduardo Vaca, Megan McNally

**Affiliations:** a University of Missouri- Kansas City School of Medicine, Kansas City, MO 64108, United States of America; b Department of Surgery, St. Luke's Health System, Kansas City, MO 64108, United States of America

**Keywords:** Retroperitoneal schwannoma, Natural progression of disease, Open resection, Case report

## Abstract

**Introduction and importance:**

Schwannomas arise from Schwann cells, which make up the neural sheath of peripheral nerves. These tumors are usually seen in the head, neck and flexor surfaces, but can arise in the retroperitoneal space on rare occasions. This case gives the unique opportunity to watch the long term progression and speed of growth of this rare tumor and the development of symptoms over time.

**Case presentation:**

Here, we describe the interesting case of a retroperitoneal Schwannoma discovered incidentally that was then monitored over the course of six years. After the tumor grew from 2.4 × 2.2 cm to 5.4 × 5.2 cm over this time, symptoms such as abdominal pain, nausea, constipation, increased urinary frequency, and left leg paresthesia arose, prompting for removal of the tumor. On follow-up two weeks later, the patient reported resolution of symptoms.

**Clinical discussion:**

Treatment for this tumor is either immediate excision or the “watch and wait” method, as this tumor has a low rate of malignant transformation. The tumor discussed in this case had a higher rate of growth before removal when compared to other studies examining retroperitoneal Schwannoma development.

**Conclusion:**

The “watch and wait” method of treatment for this benign tumor is effective, but it is important to ensure the patient is aware that the tumor will likely continue to grow. Given this, the patient should be informed of possible mass effect symptoms to monitor for.

## Highlights

• Schwannomas rarely arise in the retroperitoneal space and are often found incidentally or with symptoms of mass effect
• This retroperitoneal Schwannoma was found incidentally and monitored for growth over a six year course
• Symptomatology of these tumors depend on the structures that it perturbs with its mass effect
• Histology of retroperitoneal Schwannomas show narrow, oblong cells with Antoni A and B areas and Verocay bodies

## 1 Introduction

Schwannomas are a classification of tumor arising from Schwann cells making up the neural sheath of peripheral nerves. While most Schwannomas are found in the head, neck and flexor surfaces of the extremities, they rarely can arise in the retroperitoneal space [1,4,6]. This location makes up only 0.5–5 % of all Schwannomas, and due to its rarity, can be a challenge to diagnose [5]. Tumors in this unique location can be asymptomatic, or present with symptoms of mass effect such as bowel or bladder issues [4]. In this case report, we discuss a patient with a retroperitoneal Schwannoma found incidentally on computed tomography (CT) who was then followed in clinic over the course of about six years. As time progressed, the patient gradually began to experience mass effect symptoms from the growing tumor, which resulted in its surgical removal in the inpatient academic hospital setting.

## 2 Methods

This work was completed in line with the SCARE criteria [13].

## 3 Case presentation

A 48 year old woman presented to the surgical oncology clinic with a chief complaint of abdominal pain, nausea, constipation, increased urinary frequency, and left leg paresthesia which had progressed over the past year.

Nearly six years before this presentation, our patient underwent a bilateral salpingectomy and hysterectomy for abnormal uterine bleeding, which was complicated by formation of a vesicovaginal fistula. This necessitated further workup with abdominal CT, which revealed an incidentally found ovoid mass measuring 2.7 × 2.3 × 4.2 cm adjacent to the left common iliac artery. Further workup with abdominal magnetic resonance imagining (MRI) demonstrated a 2.4 × 2.2 cm ovoid left common iliac nodule with intermediate soft tissue signal intensity. The patient underwent a needle biopsy of the mass several months later which demonstrated a spindle cell neoplasm with low cellularity, very low mitotic activity, and no significant nuclear pleomorphism. The specimen stained positive for S100 and was identified as a Schwannoma. The patient was recommended to follow with neuro-oncology for continued surveillance of the Schwannoma.

**Fig. 1:**
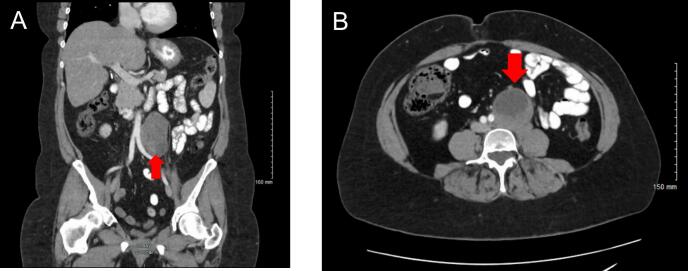
CT views of the abdomen **A:** Coronal section of the abdomen showing soft tissue density **B:** Transverse section of the abdomen showing soft tissue density.

Years later, the patient presented to surgical oncology with abdominal pain, nausea, constipation, increased urinary frequency, and left leg paresthesia as described in paragraph one. The patient's most recent CT from several months ago showed that the Schwannoma had grown in size to 5.4 × 5.2 cm without evidence of metastatic disease in the abdomen (Fig. 1). Due to the new onset of symptoms and the continued growth of the mass, surgical resection was recommended. An open procedure was performed due to the mass's size and proximity to vital structures. The case began with preoperative placement of a left ureteral stent by Urology. Next, a midline laparotomy incision was made to gain entry into the abdomen. Upon inspection, the mass was immediately felt in the left retroperitoneum. The left colon was mobilized and reflected medially to gain exposure of the left retroperitoneum and the mass. The mass was encircled medially by the aorta, inferiorly by the left iliac artery, and laterally by the left ureter. The mass did not invade into any of these structures, which allowed for it to be carefully separated. Meticulous dissection was performed until the mass was circumferentially freed without causing injury to any major vessels or structures. Once this was completed, the mass was removed in its entirety and marked for pathology.

**Fig. 2:**
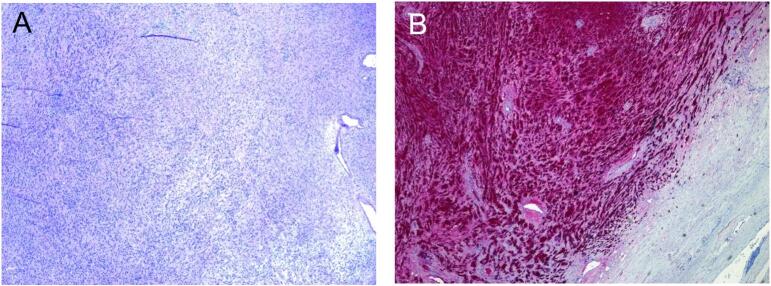
Histological views of the tumor after excision **A:** Histological view at 10x showing Antoni A and Antoni B areas. **B:** Immunohistochemical stain with S100 is diffusely positive.

The mass was then sent to pathology, which grossly showed a tan-red, ovoid soft tissue mass with sectioning revealing a tan to pale yellow, focally cystic well circumscribed mass. Histological analysis demonstrated palisading spindle shaped cells without nuclear atypia and the presence of Antoni A and B areas, as well as Verocay bodies. Staining for S100 was positive and the final diagnosis was determined to be a benign Schwannoma (Fig. 2).

During the two week postoperative follow up visit, the patient reported that the preoperative abdominal pain had since resolved. The patient will have a surveillance MRI six months after the surgery to assess for any recurrence of the mass.

## 4 Discussion

Asymptomatic retroperitoneal Schwannomas are often discovered incidentally on imaging, and can become symptomatic due to mass effect as they continue to grow [6]. These benign tumors can manifest as pressure, poorly localized abdominal pain, or issues with digestion, such as constipation [6]. Additionally, urinary symptoms and low back pain can occur, as well as a palpable mass if the tumor becomes large enough [[1], [2], [3]]. The lack of symptoms specific to Schwanommas, as well as laboratory values often within normal limits, make it a diagnosis based on exclusion, one often aided by imaging [6].

The role of biopsy in retroperitoneal masses is somewhat controversial. CT guided needle biopsy is a viable option; however, concern for damage of surrounding structures, hemorrhage, infection, or tumor seeding exists [2]. Additionally, retroperitoneal Schwanommas often have a large differential diagnosis e.g., pheochromocytoma, fibrosarcoma, and gastrointestinal stromal tumors (GIST), the latter of which biopsy is discouraged [1,3]. However, if imaging is concordant with a benign tumor and the patient is asymptomatic, then biopsy may be warranted to decide if surgery is needed or not based on the histological structure of the tumor [3,12].

Treatment of retroperitoneal Schwannoma depends largely on the symptoms experienced by the patient. For symptomatic patients, surgical resection is the mainstay of treatment, as chemotherapy and radiation are not effective on this tumor type [1,2]. In asymptomatic patients, the “wait and see” approach is reasonable given the benign nature of this tumor and the low risk of malignant transformation [1,10]. For patients who opt not to have surgical management, the natural progression of retroperitoneal Schwannomas can be observed. A retrospective study done by Ogose et al. (2019) examined 22 cases of retroperitoneal Schwannomas and found the average growth rate of the tumor to be 1.9 mm/year with a range of −1.9 mm to 8.7 mm [10]. Another study by Kitagawa et al. (2020) showed eight cases with remarkably similar results, the growth rate here averaging at 1.9 mm/year as well [11]. The case of retroperitoneal Schwannoma discussed in this case grew from 2.4 × 2.2 cm to 5.4 × 5.2 cm over a course of 5.75 years, which calculates to a growth rate of 5.2 mm/year. This is an above average rate of growth when compared to the two studies discussed above, and resulted in tumor excision due to mass effect, providing further insight on the natural progression of these tumors.

Final diagnosis of retroperitoneal Schwannoma is made based on the histological structure of the tumor. Generally, cells have a narrow, oblong and wavy appearance to their nuclei. One specific aspect of cellular architecture found in Schwannomas is the presence of Antoni A and Antoni B areas [9]. Antoni A areas are characterized by fibrillary, elongated nuclei that appear more crowded, creating an area of deeper color on low magnification [9]. In contrast, the Antoni B areas adjacent to Antoni A areas are looser, less densely packed sections that appear lighter on low magnification [9]. Verocay bodies are another aspect of Schwannomas that can be seen, and are defined as nuclear palisading around a fibrillary core [8]. More plainly, this feature has a striped appearance due to the alternating of low and high density areas of nuclei. Schwannomas most notably stain positive for S100, as seen in this case [7].

Long term follow up is necessary for patients with retroperitoneal Schwannomas as local recurrence can occur in about 5–10 % of cases, most often due to incomplete excision [1,2]. After resection, the overall prognosis of these tumors is good with recurrence being the most common complication [2].

## 5 Conclusion

Retroperitoneal Schwannomas are rare tumors that can present with mass effect symptoms or be found incidentally on imaging. The management for these tumors is either the “wait and see” approach or surgical resection, based on the patient's symptoms and goals of treatment. Imaging can lend insight to the type of tumor, but histological analysis is the gold standard for diagnosis. These tumors often continue to enlarge at a variable rate and can lead to mass effect, as seen in this case report. Retroperitoneal Schwannomas have a low risk of malignant progression. These tumors have a good long term outcome, and often only require monitoring for recurrence.

## Author contribution

Elizabeth Kruse- Conceptualization, Writing - Original Draft, Writing - Review & Editing, Project Administration.

Allye Gardner- Writing - Original Draft, Writing - Review & Editing.

Eduardo Vaca MD- Writing - Review & Editing, Supervision.

Megan McNally MD- Writing - Review & Editing, Supervision.

## Consent

Written informed consent was obtained from the patient for publication of this case report and accompanying images. A copy of the written consent is available for review by the Editor-in-Chief of this journal on request.

## Ethical approval

IRB review and approval was waived for this case report.

## Guarantor

Elizabeth Kruse

Allye Gardner

Eduardo Vaca MD

Megan McNally MD

## Sources of funding

No funding was provided for the completion of this manuscript.

## Declaration of competing interest

There are no conflicts of interest to disclose.
